# Role of Dual Pathway Inhibition in Secondary Prevention of Coronary Artery Disease

**DOI:** 10.7759/cureus.80504

**Published:** 2025-03-13

**Authors:** Fraz Ahmad, Ali Husnain, Muhammad Fakhar Hayat, Muhammad Abdullah Ashraf, Muhammad Ammar Arif, Muhammad Zarrar A Butt, Amna Junaid Qureshi, Muhammad Hamza

**Affiliations:** 1 Cardiology, Shalamar Hospital, Lahore, PAK; 2 Cardiology, Fatima Memorial Hospital, Lahore, PAK; 3 Cardiology, Fatima Memorial Hospital College of Medicine and Dentistry, Lahore, PAK; 4 Internal Medicine, Shalamar Hospital, Lahore, PAK; 5 Internal Medicine, Combined Military Hospital, Muzaffarabad, PAK

**Keywords:** acute coronary syndrome, cardiovascular risk, coronary artery disease, dual-pathway inhibition, single-pathway antiplatelet therapy

## Abstract

Introduction

Coronary artery disease (CAD) continues to be a significant global health challenge, contributing to high rates of morbidity and mortality despite advancements in medical care. Dual-pathway inhibition (DPI) represents a major advancement in the secondary prevention of CAD by targeting both platelet aggregation and thrombin generation. Unlike traditional single-pathway antiplatelet therapy (e.g., aspirin or P2Y12 inhibitors), DPI provides a synergistic approach to reduce the residual cardiovascular risk that persists despite optimal medical therapy.

Objective

To evaluate the efficacy and safety of DPI compared to standard single-pathway antiplatelet therapy in reducing cardiovascular events in patients with CAD.

Methodology

This prospective observational cohort study was conducted at Shalamar Hospital, Lahore, Pakistan, from September 2023 to August 2024. Data were collected from 147 patients diagnosed with stable CAD or who had experienced a recent acute coronary syndrome. Patients were randomly assigned into two groups: the DPI group and the control group. The DPI group (n=74) received a combination of low-dose rivaroxaban (2.5 mg twice daily) and aspirin (81 mg daily), while the control group (n=73) received standard single-pathway antiplatelet therapy with aspirin (81 mg daily).

Results

The mean age was similar in both groups, with 56.67 ± 8.01 years in the DPI group and 58.92 ± 9.23 years in the control group (p=0.48). The proportion of male patients was comparable, with 50(68%) in the DPI group and 48(66%) in the control group (p=0.78). Prevalence rates of hypertension [53(72%) vs. 54(74%), p=0.74], diabetes mellitus [31(42%) vs. 29(39%), p=0.69], and previous myocardial infarction (MI) [35(48%) vs. 37(51%), p=0.66] were nearly identical across groups. Similarly, smoking history showed no significant difference, with 26(35%) in the DPI group and 24(33%) in the control group (p=0.81). Cardiovascular deaths were less frequent in the DPI group [2(2.7%)] compared to the control group [5(6.8%)], although this difference did not reach statistical significance (p=0.18). Similarly, the incidence of non-fatal MI [3(4.1%) vs. 7(9.6%), p=0.12] and non-fatal stroke [2(2.7%) vs. 3(4.1%), p=0.63] was lower in the DPI group but not statistically significant.

Conclusion

It is concluded that DPI is an effective strategy for reducing residual cardiovascular risk in patients with CAD. By combining platelet aggregation and thrombin generation inhibition, DPI significantly lowers the incidence of major adverse cardiovascular events compared to single-pathway antiplatelet therapy.

## Introduction

Coronary artery disease (CAD) continues to be a significant global health challenge, contributing to high rates of morbidity and mortality despite advancements in medical care. CAD develops because atherosclerotic plaques form in coronary arteries, causing events of ischemia such as myocardial infarction (MI) and angina. Secondary prevention of CAD is directed at reducing further cardiovascular events, which have significant rates despite the optimal medical management of the condition. The above-mentioned residual risk can be minimized by the new dual-pathway inhibition (DPI), which is the approach to target the multi-mechanistic nature of thrombosis and inflammation [1]. DPI represents a major advancement in the secondary prevention of CAD by targeting both platelet aggregation and thrombin generation. Unlike traditional single-pathway antiplatelet therapy (e.g., aspirin or P2Y12 inhibitors), DPI provides a synergistic approach to reduce the residual cardiovascular risk that persists despite optimal medical therapy. Typical secondary prevention in CAD has primarily been stream-based, relying solely on antiplatelet therapy with aspirin or P2Y₁₂ inhibitors. Though these therapies are helpful in minimizing the risks of arterial thrombosis, they are generally inadequate to manage the variety of etiologic aspects of CAD [2]. That’s why residual cardiovascular risk still rises partly due to the activation of other processes, such as the coagulation cascade or inflammation. This underlines the necessity to develop and apply more complex therapeutic strategies, including DPI, which involves two mechanisms: the interference with platelet aggregation and the modulation of thrombin generation, to ensure synergistic protection against thromboembolic events [3]. Clinical studies of DPI involve using two distinct pharmacological processes to enhance the prognosis of patients with CAD [4]. The first mechanism includes the reduction of platelet aggregation, which plays an active role in arterial thrombosis. Platelet aggregation mostly occurs by the activation of GPIIb/IIIa receptors and a P2Y12 receptor resulting in the formation of platelet clots. Antiplatelet drugs include aspirin, a COX-1 (cyclooxygenase-1) blocker, or P2Y12 blockers such as clopidogrel, ticagrelor, and prasugrel [5]. The second mechanism in DPI is the prevention of clot formation and subsequent inhibition of thrombin generation in the coagulation scheme. Tissue activation at the site of the thrombus is carried out by thrombin, a potent enzyme involved in the transformation of fibrinogen to fibrin, stabilization of the thrombus, and activation of platelets [6]. Paradoxically anticoagulation is used by targeting specific coagulation proteins with factor Xa inhibitors (such as rivaroxaban) or direct thrombin inhibitors (such as dabigatran) to suppress thrombin activity and prevent clot formation. A number of published clinical trials have assessed the use of DPI as a method to assess its efficiency and safety in patients with CAD. The largest such study ever conducted, the COMPASS trial, established that rivaroxaban 2.5 mg and 5 mg with aspirin can reduce major adverse cardiovascular events (MACEs) by 24% compared to aspirin alone for stable atherosclerotic vascular disease. This trial established new possibilities for DPI to deliver a higher level of secondary prevention in individuals at high risk [7].

There was also the ATLAS ACS 2-TIMI 51 comparison of low-dose rivaroxaban in the treatment of acute coronary syndrome (ACS). The study highlighted reductions in cardiovascular deaths, MI, and stroke, although it was associated with a higher rate of bleeding. Suffice to note that these results emphasize the need to maintain the optimal balance of efficiency and safety in the practical use of DPI [8]. However, DPI has some drawbacks, mainly concerning bleeding risk, though it presents substantial advantages in many respects compared to conventional inhaler therapies. The finding suggests that combining antiplatelet agents with antithrombotic agents entails a higher risk of both major and minor bleeding and thus requires considerate patient selection and risk profiling. When prescribing DPI, priorities should be given to patients’ age, comorbidities, renal functions, and concurrent medications, as they may affect its results [9]. Future developments in DPI precision delivery systems and pharmacogenomic knowledge will enable clinicians to optimize DPI regimens and enhance heir safety for certain patient populations. DPI is a major step forward in the secondary prevention of CAD because it targets the major factors resulting from platelet aggregation and thrombin generation [10]. DPI has the potential to synergistically manage thrombosis and inflammation, which if deployed could revolutionize the future of patients with high-risk CAD. Nevertheless, it should always be implemented with special consideration for bleeding risk, patient selection, and cost-benefit analysis. As the understanding of DPI as a research topic advances and is applied in more specific ways, there is optimism in the end that it will ultimately help offload the world’s burden of CAD and enhance the quality of life for millions of patients across the globe [11].

This study evaluates the effectiveness and safety of DPI compared to single-pathway antiplatelet therapy in preventing MACEs in patients with high-risk CAD.

## Materials and methods

This prospective observational cohort study was conducted at Shalamar Hospital, Lahore, Pakistan, a tertiary care center recognized for its high volume of cardiovascular procedures, particularly in managing CADs. Patients were enrolled from September 2023 to August 2024. Data were collected from 147 patients diagnosed with stable CAD or who had experienced a recent ACS.

Eligible participants were adults aged 40-75 years, with confirmed CAD or a recent ACS. They were required to have a high risk of recurrent cardiovascular events, such as a history of MI, multi-vessel CAD, or peripheral artery disease. Only clinically stable individuals who could tolerate aspirin and anticoagulants were eligible.

Participants were excluded if they had active bleeding or a major bleeding episode within the past six months. Severe liver dysfunction (Child-Pugh B/C) or significant renal impairment (estimated glomerular filtration rate <30 mL/min/1.73 m²) also led to exclusion. Those with hypersensitivity to the study drugs or requiring therapeutic anticoagulation outside the study protocol were not eligible. Additionally, individuals with planned major surgery, pregnancy, or a life expectancy of less than one year due to non-cardiovascular conditions were excluded.

Patients were randomly assigned into two groups: the DPI group and the control group. The DPI group (n=74) received a combination of low-dose rivaroxaban (2.5 mg twice daily) and aspirin (81 mg daily), while the control group (n=73) received standard single-pathway antiplatelet therapy with aspirin (81 mg daily).

The principal interest endpoint was the rate of the MACEs that encompassed cardiovascular death, non-fatal MI, and non-fatal stroke. The main safety outcome was major and clinically relevant non-major bleeding events, based on the International Society on Thrombosis and Hemostasis (ISTH) definitions. 

Data were prospectively collected through a combination of electronic medical records and structured patient interviews. Baseline characteristics, including age, sex, body mass index, comorbidities (e.g., hypertension, diabetes, chronic kidney disease), smoking status, prior cardiovascular events, medication history, and socioeconomic factors, were recorded to account for potential confounders. Patient follow-up occurred at baseline, one month, six months, and twelve months, with comprehensive clinical examinations and biochemical tests conducted for both safety assessment and efficacy evaluation. Biochemical testing included full blood counts, prothrombin time, activated partial thromboplastin time, international normalized ratio, and liver function tests. These tests were performed at each follow-up visit to monitor coagulation status, hepatic function, and potential drug-related adverse effects. Standardized laboratory protocols ensured consistency and abnormal results were reviewed by clinicians for necessary interventions. Medication adherence was assessed through a multi-faceted approach. In addition to patient self-reports via diaries, adherence was objectively evaluated by pill counts at each follow-up visit. To enhance accuracy, pharmacy refill data were also considered. Any discrepancies between self-reports and objective measures were discussed with patients to identify potential adherence barriers and improve compliance.

Data were analyzed using SPSS Version 26.0 (IBM Corp., Armonk, NY). Categorical variables were summarized as frequencies and percentages, while continuous variables were presented as means and standard deviations. Chi-square tests were employed for categorical comparisons, and Independent t-tests were used to compare continuous variables. A multivariate COX regression analysis was conducted to identify outcomes, with hazard ratios (HR) and 95% confidence Interval reported. All statistical tests were two-tailed, and a p-value of <0.05 was considered statistically significant. This methodology provided a comprehensive framework to evaluate the efficacy and safety of DPI in the secondary prevention of CAD.

The study was conducted in accordance with the ethical standards of the Institutional Review Board of Shalamar Medical and Dental College, Lahore, which granted approval for the research (approval number: SMDC-IRB/AL/28/2023). Written informed consent was obtained from all participants, and patient confidentiality was rigorously maintained throughout the study. Data were anonymized before analysis to ensure privacy.

## Results

Data were collected from 147 patients. The mean age was similar in both groups, with 56.67 ± 8.01 years in the DPI group and 58.92 ± 9.23 years in the control group (p=0.48). The proportion of male patients was comparable, with 50(68%) in the DPI group and 48(66%) in the control group (p=0.78). Prevalence rates of hypertension [53(72%) vs. 54(74%), p=0.74], diabetes mellitus [31(42%) vs. 29(39%), p=0.69], and previous MI [35(48%) vs. 37(51%), p=0.66] were nearly identical across groups. Similarly, smoking history showed no significant difference, with 26(35%) in the DPI group and 24(33%) in the control group (p=0.81) (Table 1).

**Table 1:** Baseline characteristics DPI, Dual Pathway Inhibition; SD, Standard Deviation p-value of <0.05 is considered statistically significant.

Characteristic	DPI Group (Total Participants=74) N (%) or Mean (SD)	Control Group (Total Participants=73) N (%) or Mean (SD)	p-Value
Mean Age (Years)	56.67 (±8.01)	58.92 (±9.23)	0.48
Male (%)	50 (68%)	48 (66%)	0.78
Hypertension (%)	53 (72%)	54 (74%)	0.74
Diabetes Mellitus (%)	31 (42%)	29 (39%)	0.69
Previous Myocardial Infarction (%)	35 (48%)	37 (51%)	0.66
Smoking History (%)	26 (35%)	24 (33%)	0.81

**Figure 1:**
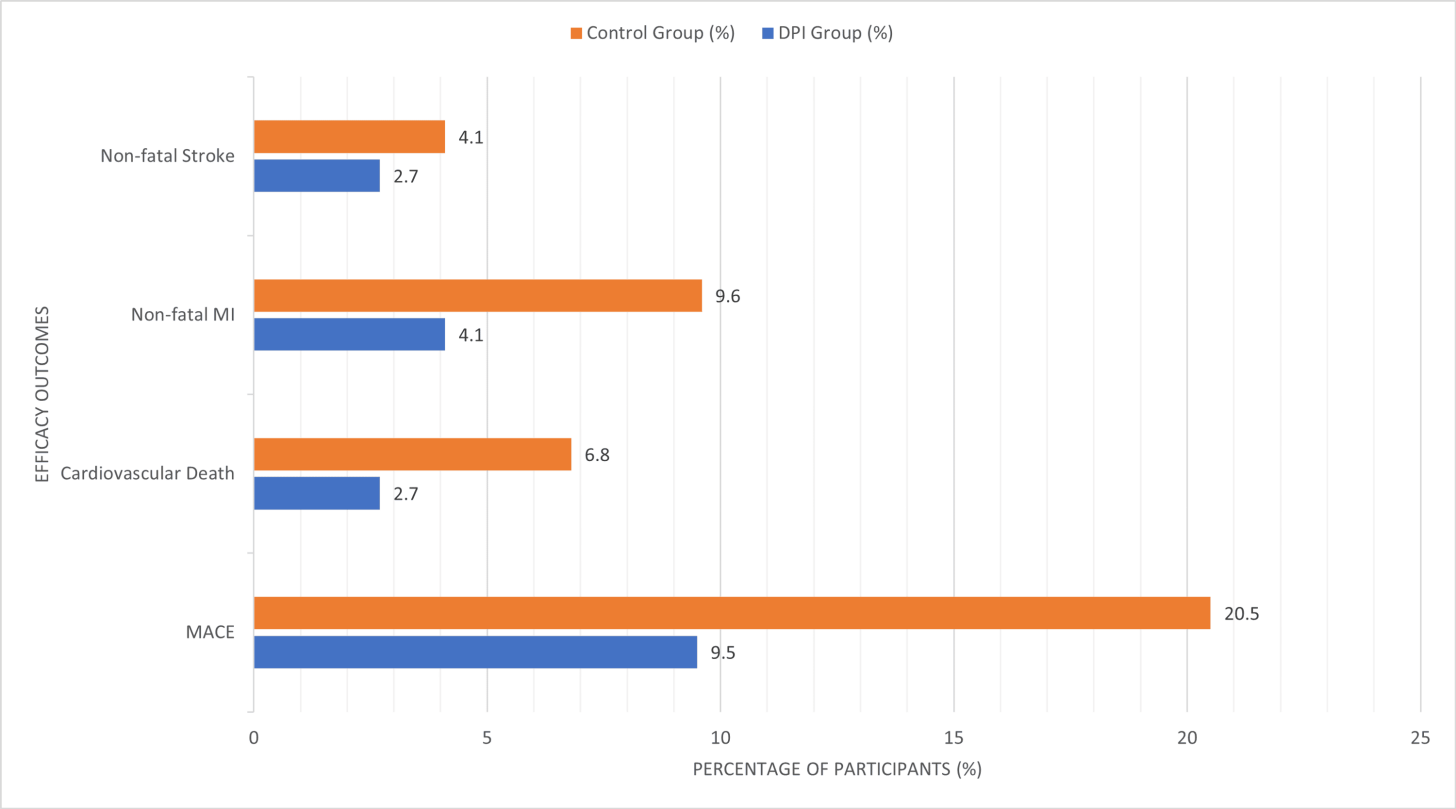
Efficacy outcomes DPI, Dual Pathway Inhibition; MACE, Major Adverse Cardiovascular Event; MI, Myocardial Infarction p-value of <0.05 is considered statistically significant.

Figure 1 demonstrated a significant reduction in MACEs in the DPI group [7(9.5%)] compared to the control group [15(20.5%)], with a p-value of 0.02 and a hazard ratio of 0.45 (95% CI: 0.23-0.88). Cardiovascular deaths were less frequent in the DPI group [2(2.7%)] compared to the control group [5(6.8%)], although this difference did not reach statistical significance (p=0.18). Similarly, the incidence of non-fatal MI [3(4.1%) vs. 7(9.6%), p=0.12] and non-fatal stroke [2(2.7%) vs. 3(4.1%), p=0.63] was lower in the DPI group but not statistically significant (Table 2).

**Table 2:** Efficacy Outcomes DPI, Dual Pathway Inhibition; CI, Confidence Interval p-value of <0.05 is considered statistically significant.

Outcome	DPI Group (Total Participants=74) N (%)	Control Group (Total Participants=73) N (%)	p-Value	Hazard Ratio	95% CI
Major Adverse Cardiovascular Events	7 (9.5%)	15 (20.5%)	0.02	0.45	0.23–0.88
Cardiovascular Death	2 (2.7%)	5 (6.8%)	0.18	—	—
Non-Fatal Myocardial Infarction	3 (4.1%)	7 (9.6%)	0.12	—	—
Non-Fatal Stroke	2 (2.7%)	3 (4.1%)	0.63	—	—

The safety analysis revealed a higher incidence of major bleeding in the DPI group [5(6.8%)] compared to the control group [2(2.7%)], although the difference was not statistically significant (p=0.15). Clinically relevant non-major bleeding was also more frequent in the DPI group [8(10.8%)] compared with the control group [3(4.1%)], approaching statistical significance (p=0.09). Importantly, no fatal bleeding events were reported in either group. Hospitalizations for unstable angina or heart failure were significantly lower in the DPI group [10(13.5%)] than in the control group [17(23.3%)], with a p-value of 0.04. Additionally, adherence to therapy was higher in the DPI group (93%) than in the control group (87%) (Table 3).

**Table 3:** Safety outcomes DPI, Dual Pathway Inhibition p-value of <0.05 is considered statistically significant.

Outcome	DPI Group (Total Participants=74) N (%)	Control Group (Total Participants=73) N (%)	p-Value
Major Bleeding	5 (6.8%)	2 (2.7%)	0.15
Clinically Relevant Non-Major Bleeding	8 (10.8%)	3 (4.1%)	0.09
Fatal Bleeding	0%	0%	—
Hospitalization for Unstable Angina/Heart Failure	10 (13.5%)	17 (23.3%)	0.04
Adherence to Therapy (%)	93%	87%	—

## Discussion

This study highlights the potential benefits of DPI in the secondary prevention of CAD, demonstrating significant reductions in MACEs compared with standard single-pathway antiplatelet therapy. The results correlate with other studies, including the COMPASS trial, which confirmed the feasibility of using low-dose rivaroxaban with aspirin to cut down on CV events. However, bleeding events were identified as having increased, which clearly emphasizes the need to select appropriate patient populations and assess risk [12]. The lower rate of MACE in the DPI group [7(9.5%)] as compared with the control group [15(20.5%)] enhances the effectiveness of targeting both platelet activation and thrombin formation. DPI thus protects against ischemic stroke by targeting two different paths in thrombus evolution. This is especially relevant in a high-risk population, including those with multi-vessel CAD or a history of MI, where residual cardiovascular risk remains far from insignificant despite the best medical management [13]. Although DPI proved effective in the case, the comparative outcomes display increased major bleeding rates of 5(6.8%) in the DPI group compared with 2(2.7%) in the placebo group. While this difference was not statistically significant in this study, the result is consistent with earlier trials where dual-pathway strategies led to more bleeding. The lack of fatal bleeding events remains promising and again underlines the concept that improvement of therapeutic benefit should be weighed against the potential harms of the adverse events [14]. The findings of this study offer evidence for DPI, along with key research considerations, as a feasible approach to secondary prevention in patients with CAD. Patients at moderate to high risk for ischemic events but with a low bleeding risk are likely to benefit from this approach [15,16]. Evaluations that should influence management decisions include patients’ age, initial renal function, and history of previous bleeding [17]. Furthermore, compliance with the therapy, which was higher in the DPI group (93%), is an important determinant for achieving the best results that may imply better patient cooperation in all-encompassing treatment plans [18]. To a certain extent, this study possesses some limitations that have to be discussed. The inherent hypothesis of the study is that the duration of DPI therapy is associated with the risk of new-onset atrial fibrillation, which is moderate at best. As a result, certain methodological constraints, such as the small sample size (147 patients) and single-center design, may weaken the findings. Additionally, since we enrolled participants for just over a year, we were unable to track the long-term prospective effects of DPI, whether beneficial or unfavorable. It will be pertinent to carry out future multi-centered trials with larger population groups and longer follow-up durations to validate these observations and to study the steady-state safety profile of DPI. Biomarkers or other measurements are being investigated to refine the use of DPI, ensuring that only appropriate patients are selected for this strategy in precision medicine. Pharmacogenomic development might make it possible to optimize dosing schedules and, at the same time. reduce rates of bleeding. Further, when DPI is combined with lifestyle modifications and other therapies, the benefits are likely to be significant in patients with CAD.

## Conclusions

In conclusion, DPI represents a promising and effective strategy for mitigating residual cardiovascular risk in patients with CAD. By simultaneously targeting platelet aggregation and thrombin generation, DPI demonstrates a significant reduction in the incidence of MACEs when compared to single-pathway antiplatelet therapy. Nevertheless, the associated increase in bleeding risk underscores the importance of meticulous patient selection and the development of individualized treatment regimens. Optimizing the balance between therapeutic efficacy and safety remains critical to maximizing the clinical benefits of DPI in this high-risk patient population. Further research is warranted to refine risk stratification and identify patients who are most likely to benefit from this approach.
